# Testing pseudotopological and nontopological models for SMC-driven DNA loop extrusion against roadblock-traversal experiments

**DOI:** 10.1038/s41598-023-35359-2

**Published:** 2023-05-19

**Authors:** Roman Barth, Biswajit Pradhan, Eugene Kim, Iain F. Davidson, Jaco van der Torre, Jan-Michael Peters, Cees Dekker

**Affiliations:** 1 Department of Bionanoscience, Kavli Institute of Nanoscience Delft, Delft University of Technology, Delft, The Netherlands; 2 Research Institute of Molecular Pathology (IMP), Vienna Biocenter (VBC), Vienna, Austria; 3 Present Address: Max-Planck Institute of Biophysics, Frankfurt Am Main, Germany

**Keywords:** Biophysics, Molecular biology

## Abstract

DNA loop extrusion by structural-maintenance-of-chromosome (SMC) complexes has emerged as a primary organizing principle for chromosomes. The mechanism by which SMC motor proteins extrude DNA loops is still unresolved and much debated. The ring-like structure of SMC complexes prompted multiple models where the extruded DNA is topologically or pseudotopologically entrapped within the ring during loop extrusion. However, recent experiments showed the passage of roadblocks much bigger than the SMC ring size, suggesting a nontopological mechanism. Recently, attempts were made to reconcile the observed passage of large roadblocks with a pseudotopological mechanism. Here we examine the predictions of these pseudotopological models and find that they are not consistent with new experimental data on SMC roadblock encounters. Particularly, these models predict the formation of two loops and that roadblocks will reside near the stem of the loop upon encounter—both in contrast to experimental observations. Overall, the experimental data reinforce the notion of a nontopological mechanism for extrusion of DNA.

## Introduction

Higher order genome organization is largely dictated by structural maintenance of chromosome complexes (SMC) complexes^1–3^. SMC complexes including condensin, cohesin, and Smc5/6 in eukaryotes, share a ring-like architecture consisting of multiple subunits were two coiled-coil SMC arms and a intrinsically disordered kleisin constitute a large (~ 35 nm) ring. Additional subunits attach to the kleisin and ATPase heads of the SMC subunits, rendering different functions to the different SMC complexes. SMC proteins organize the chromosome by DNA loop extrusion, as demonstrated by single-molecule in vitro experiments^4–6^. However, the mechanistic understanding of the coordinated movement of SMC subunits that underlie loop extrusion is still in its infancy.

The topology of DNA involved during loop extrusion has been a major point of discussion in recent years^1,7–9^. Three possibilities have been proposed: (i) topological binding, where the SMC ring opens to encircle the DNA, (ii) pseudotopological loading, where a small nascent loop enters the SMC ring without opening, and where loop extrusion continues pseudotopologically by further extruding this inserted loop through the ring, (iii) nontopological loading where the DNA does not enter the ring to extrude loop but instead binds at the outer interfaces of the SMC ring.

Recently, we showed that condensin and cohesin can bypass very large (even 200 nm) DNA-bound proteins and particles while extruding DNA loops^10^. Upon encounter of such a DNA-bound particle by the loop-extruding SMC complex, the particle transfers into the extruded DNA loop in the majority of cases. We even observed such passage of large particles by single-chain cohesins where all interfaces of the SMC subunits and the kleisin were covalently linked^5^. The passage of roadblocks bigger than the ring size of cohesin, even when the ring cannot open, clearly indicated that the extruded DNA does not enter the ring topologically or pseudotopologically. Instead, these experiments pointed to a nontopological mechanism for loop extrusion.

After these data appeared on bioRxiv in July 2021^10^, two new pseudotopological models were proposed by Shaltiel et al*.* (“hold-and-feed mechanism^11^”) and by Nomidis et al*.* (“segment-capture model^12^”). In the light of the roadblock experiments, the authors of these models pursued to explain the passage of the huge roadblocks within the context of their models.

Here we provide a detailed evaluation of these models with regards to the passage of large DNA-bound roadblocks upon encounter with a loop-extruding SMC complex. We spell out particular predictions that result from these models and evaluate whether or not these predictions are confirmed by experimental observations. We show that the predictions are not consistent with the experimental data for encounters of a loop-extruding SMC complex with DNA-bound roadblocks. The analysis points to a nontopological mechanism instead of a pseudotopological model for loop extrusion.

## Results

### Description of the pseudotopological models

According to the pseudotopological models proposed by Shaltiel et al*.*^11^ and Nomidis et al*.*^12^, SMC complexes entrap DNA pseudo-topologically, i.e. the two DNA strands that form the base of a loop pass through the ring structure formed by the SMC and kleisin subunits of these complexes. In view of the clear observation of the passage of roadblocks bigger than the SMC ring size into extruded loops in our experiments, the authors of these papers attempted to explain our observations within the context of their models. In other words, they provided an alternative interpretation of our data reported in Ref.^10^. The authors of the papers argued that their model was not necessarily inconsistent with our observation that large roadblocks are able to enter extruded loops, even though these roadblocks are larger than the ring size of SMC complexes. If they are correct, it would question our conclusion that the data point to a nontopological mechanism for DNA loop extrusion by SMCs. Below, we evaluate this alternative explanation in the light of our experimental observations.

**Figure 1:**
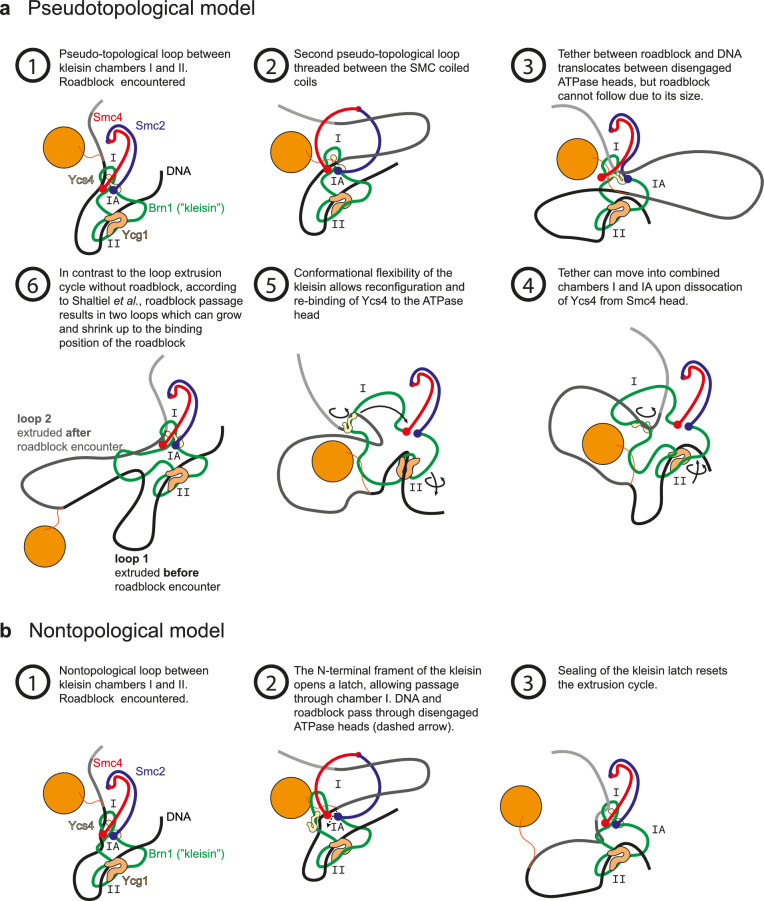
Description of the mechanism postulated by Shaltiel et al. for roadblock passage into an extruded loop on the DNA and a potential nontopological model. (**a**) The steps through the proposed DNA loop extrusion cycle are commented in more detail in steps 1–6 within the figure. Adapted from Ref.^11^. (**b**) Potential nontopological model which is closely analogous to the pseudotopological model, but with a slight variation in the DNA-SMC topology which allows particle bypass.

First, let us summarize the alternative model that Shaltiel et al*.* and Nomidis et al*.* proposed^11,12^. We focus on the model of Shaltiel et al*.* who most explicitly pointed out the dynamics involved in the roadblock passage, according to their model, but the same considerations apply to the model by Nomidis et al*.* Without explicitly commenting on the detailed SMC conformational changes involved in these models (which also differ between these models), we describe how these authors pursue to explain the passage of roadblocks bigger than the ring size of SMCs. Figure 1a illustrates the model for roadblock passage by Shaltiel et al*.* Briefly, the model for loop extrusion involves the trapping of DNA in three chambers (I, IA, and II) in yeast condensin, where extrusion is proposed to proceed through a concerted action called hold-and-feed mechanism. DNA binding to Ycg1 and the associated kleisin region act as a “safety belt”. DNA loading into chambers I and II initiates extrusion of DNA. Two pseudo-topologically entrapped DNA loops are thus formed; one between chambers I and II, and the second one threaded between the SMC coiled coils. Upon ATP hydrolysis, these two pseudo-topological loops merge into one loop (loop-1), and the SMC starts extrusion of the next segment of DNA. Note that the figure illustrates only tiny loops, whereas in our experiments the encounter of a large roadblock with a DNA-extruding SMC is observed when the loop has been already significantly extended. Specifically, this means that the loops sketched in most panels of the figures will in practice be a very large loop of many kbp of DNA in our experiments.

For comparison, Fig. 1b depicts a potential nontopological model which is consistent with the crosslinking data by Shaltiel et al*.* while it also allows roadblock passage^8^. The basic mechanism is analogous to the pseudotopological model of Shaltiel et al. in the sense that a small DNA loop is temporarily reeled through the Smc lumen, triggered by binding of DNA on the engaged ATP heads. The N-terminal kleisin however can latch out which allows traversal of a DNA-bound protein through chamber I. Disengaging the ATP heads allows the roadblock to merge with the large, previously extruded, loop. Sealing of chamber I resets the loop extrusion cycle.

Rather than commenting on the plausibility of the various steps in these models, we here make predictions of what should be observed when a roadblock particle is encountered in the case that the pseudotopological model would hold. Subsequently we turn to the experimental observations and evaluate whether or not these predictions are confirmed by the observations.

### Predictions by the pseudotopological models

Specifically, the alternative models make the following predictions for the roadblock experiments:

*Prediction 1: Appearance of two loops in side-flow visualization.*

and

*Prediction 2: Roadblocks will reside near the stem of the loop upon encounter.*

**Figure 2:**
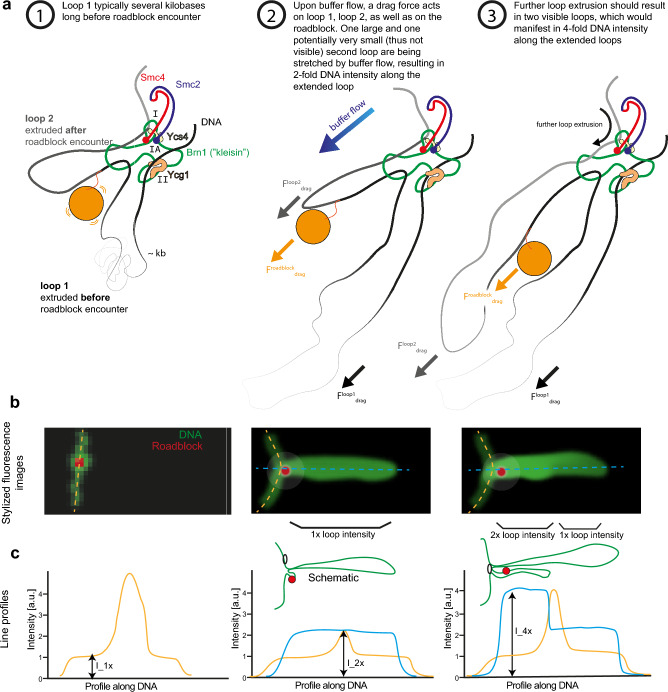
Two (not one) loops are associated with the pseudotopological model. (**a**) Presence of two loops during side-flow. (**b**) Predicted stylized intensity images (not real experimental data) of the double tethered DNA (green) with the roadblock (red). (**c**) Expected intensity profiles along the dashed lines in the images of panel (**b**).

When condensin encounters a large roadblock, the model predicts the existence of *two* loops (Fig. 2a), not one, since the roadblock—with its size that cannot pass the ring structure—obstructs the merging of the two preformed loops described above. Next to the already formed extruded loop, a new second loop is therefore formed, where the latter loop has the roadblock particle attached.

**Figure 3:**
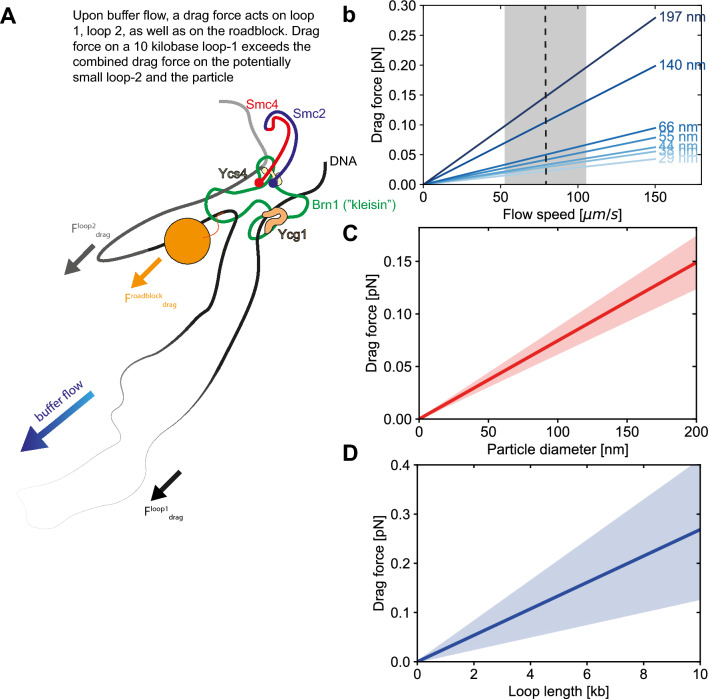
Drag forces on loops and particle associated with the pseudotopological model. (**a**) Schematic showing the blockage of the roadblock in presence of a large loop-1. (**b**) Estimated drag forces acting on particles of different sizes. The dashed line and grey area denote the experimentally measured flow speed of 79 ± 26 μm/s (N = 20). (**c**) The drag force on the roadblock (red, standard deviation based on uncertainty in buffer flow speed) versus particle size at the experimentally measured flow speed. (**d**) Drag force on DNA loop versus loop length (blue line and shaded area denote mean and standard deviation, respectively. Standard deviations are based on the uncertainty in buffer flow speed (panel (**b**)) and drag coefficient^13^.

One could assume (as Shaltiel et al*.* appear to do without further consideration) that the preformed loop-1 might slip into loop-2, to again form 1 joint loop. This however is not the case, as can be seen from considering the force balance (Fig. 3a): If side-flow is applied, several forces in action can be distinguished:

(i) A drag force on the particle ${F}_{drag}^{roadblock}=6\pi \eta Rv$, where the viscosity is $\eta =1~mPa s$ for a water-based buffer, $R$ is the particle radius, and $v$ the buffer flow speed. We experimentally measured the flow speed in side-flow experiments from occasionally observed beads that were not attached to DNA and were flowing through the field of view. We measured a flow speed of 79 ± 26 µm/s (n = 3 independent experiments). The observed forces for different particle sizes are shown in Fig. 3b,c and the values are below 0.15 pN for the largest 200 nm particles, and smaller for smaller roadblocks, down to 0.02 pN for a 30 nm particle.
(ii) Drag force on loop-1 (${F}_{drag}^{loop-1}$). This can be estimated by calculating the tension on a flow-stretched DNA, as described by Pederson et al*.*^13^ (see “Materials and methods”). Practically in our experiments, by the time that a roadblock encounters condensin, the size of loop-1 is observed to typically exceed 10 kilobases, which is associated with a drag force of value more than 0.27 pN (Fig. 3c).
(iii) Drag force on loop-2 (${F}_{drag}^{loop-2}$). As loop-2 is a very small nascent loop (~ 100 bp), the force acting on it is initially negligible, but it may become more significant as the loop continues to grow. The method to calculate the drag force acting on loop-2 is then analogous to loop-1.

It follows that upon encounter the drag force on loop-2 is nihil, while the drag force on loop-1 dominates and is larger than the drag force on the particle. As a result, the pre-encounter-formed loop-1 remains stable, while the particle remains localized close to condensin.

As loop extrusion proceeds, loop-2 grows and is slowly stretched alongside loop-1. Because of limitations in optical resolution, it is challenging to mutually distinguish the two loops spatially. However, the *intensity* of the two loops can be easily distinguished from that of a single loop by drawing intensity profiles across the loop during side-flow. Figure 2b and c illustrate what, according to the pseudotopological model, would be expected in stylized intensity images and intensity profiles along the DNA for the pre-encounter and post-encounter loops. In summary, the pseudotopological model predicts the observation of two loops that should be observed in a double intensity of that of the pre-encounter-formed loop-1 (prediction 1). The nontopological model instead predicts that the roadblock can be passed by SMC complexes, and accordingly only one loop forms (Fig. 1b).

Furthermore, the pseudotopological model predicts that the roadblock, resides at the stem of the loop after encounter (prediction 2), while the second loop keeps growing. It is of interest to note two features connected to this prediction: First, the effects of spontaneous Brownian motion can be estimated to be small. $F=-\Upsilon v+\mathrm{\delta F}(\mathrm{t})\approx 1 fN$; where $\Upsilon = 6\mathrm{\pi \eta R}$, η is dynamic viscosity, R the radius of the particle, and the typical velocity of a 10 nm particle is conservatively estimated at its upper bound, which is ~ 1 μm/s for an active motor^14^. The fluctuating force is on average zero, $\langle \mathrm{\delta F}(\mathrm{t})\rangle = 0$, but the autocorrelation function can be given as $\langle \mathrm{\delta F}(\mathrm{t})\mathrm{\delta F}(\mathrm{t}+\uptau )\rangle 2\mathrm{\Upsilon kT\delta }(\uptau )$, which leads to $\langle {\mathrm{\delta F}(\mathrm{t})}^{2}\rangle 2\mathrm{\Upsilon kT}\approx (1{\mathrm{fN})}^{2}$. The Brownian forces to move the particle away from the SMC are thus very low, in the order of a few fN, so an appreciable spontaneous drifting away of the particle is very unlikely. Second, on long time scales, when the second loop would grow to a size comparable to the first loop (~ 10 kbp), the drag force on the particle plus that on loop-2 may exceed the drag force on loop-1, which may initiate a slipping process. However, this would occur only rarely in our assay due to the finite total length of the DNA which limits the overall growth of the loop. Indeed, the typical loop size of loop at the moment of encounter (i.e. loop-1) was on average 12 kb in our experiments, while the added amount of DNA after that (i.e. loop-2 in the pseudotopological model) was 6 kb on average.

Summing up, according to the pseudotopological model, one would expect that the roadblock resides at the stem of the loop after encounter (prediction 2). In contrast, only one loop is predicted to appear in the nontopological model (Fig. 1b), and the roadblock is expected to move away from the stem of the loop as the loop continues to grow.

*Prediction 3: Loop size will increase while the mean squared displacement may stay constant after roadblock encounter*

**Figure 4:**
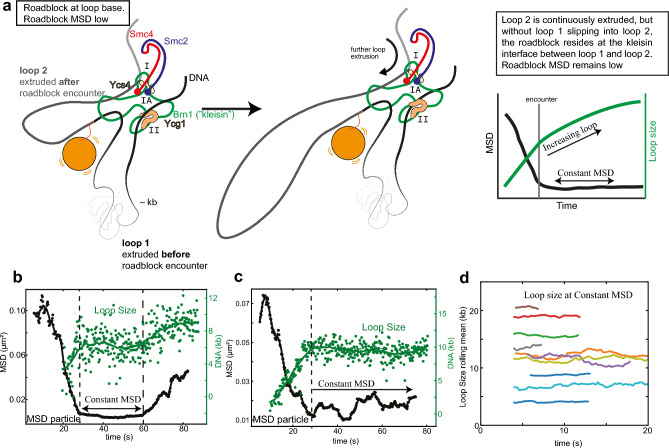
Test of prediction 3 associated with the pseudotopological model. (**a**) Correlation between DNA loop size and MSD of the particle after the encounter between condensin and roadblock, associated with the pseudotopological model. (**b,c**) Examples of kinetics of MSD (black) and loop sizes of extrusion (green) for events where the MSD remained low for a longer duration. (**d**) Rolling mean of loop size with a window size of 20 points as a function of time in events where the MSD remained constant. Different colors represent different DNA molecules (N = 10). λ-DNA and a gold nanoparticle of 39 nm was used in all panels.

What happens when the loop extruding SMC encounters a roadblock on DNA? We note that the hold-and-feed model is not considering that loop extrusion will be blocked in this case, but rather that the roadblock ends up in a second loop. In the absence of side flow, upon encounter with the roadblock (as explained in the previous point), loop-2 with the roadblock particle grows through extrusion of DNA, but the particle stays close to the SMC due to the higher drag force on loop 1 than on the particle. This makes a specific prediction for the observation of simultaneously a continued growth of the loop size without, however, any significant increase in the mean squared displacement (MSD) of the particle, as illustrated in Fig. 4A. The nontopological model instead predicts that the roadblock is in the single extruded loop which enlarges further upon extrusion after roadblock passage (see previous point). Hence, as the loop grows, the MSD of the particle is expected to grow.

### Experimental tests of the predictions made above

Our previous study^10^ presented an abundant set of experimental observations of encounters of a loop-extruding SMC complex and a DNA-bound roadblock. Here, we test whether or not the above predictions of the pseudotopological model are confirmed by the experimental observations. For the latter, we refer to the published data^10^, but we here also present additional new data that were all acquired with the same methodology and under the same conditions as in Ref.^10^.

#### Test of prediction 1

**Figure 5:**
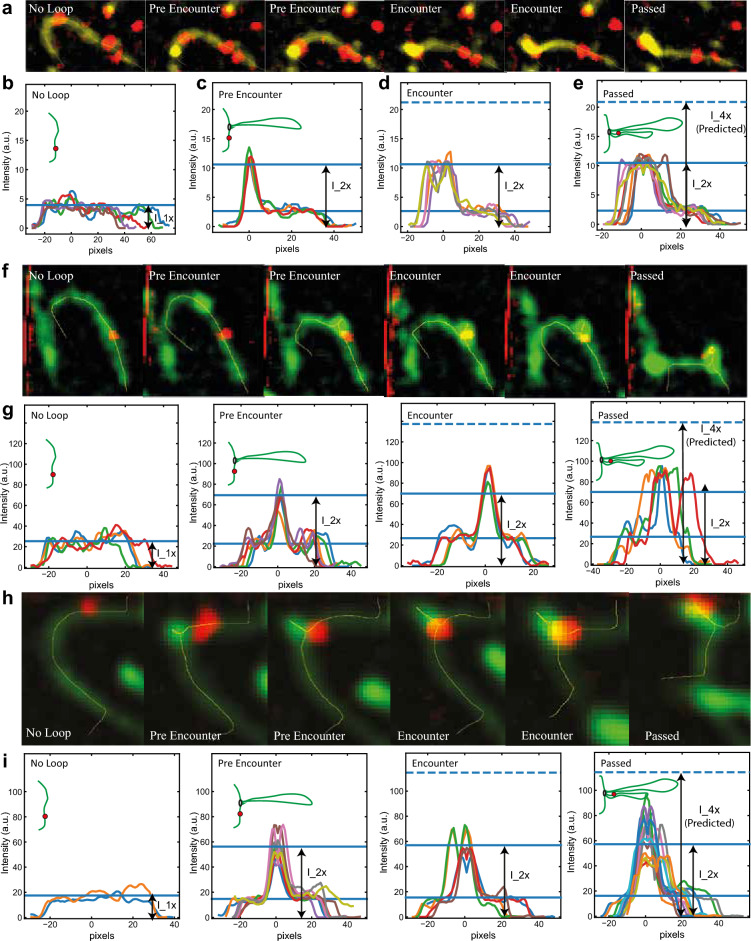
Test of prediction 1 associated with the pseudotopological model. (**a**) Snapshots of DNA and roadblock with sideflow representing different scenarios of intensity profiles in B-D namely No Loop, Pre Encounter, Encounter, and Passed the roadblock. Intensity profiles of DNA with a roadblock under sideflow before loop extrusion ((**b**), No Loop), with loop but before encountering roadblock ((**c**), Pre Encounter), at encounter when the roadblock is at the stem of the loop ((**d**), Encounter), and after the encounter when the roadblock is inside the loop ((**e**), Passed). Different colors represent different frames. (**f–i**) Two more examples of the intensity profiles on DNA at different stages of the loop extrusion. λ-DNA and a gold nanoparticle of 39 nm was used in all panels.

To test whether or not the extruded DNA forms one or two loops when a roadblock is encountered, we measured intensity profiles of DNA at different stages of loop extrusion after a roadblock encounter, see Fig. 5. The intensity of a single loop (Fig. 5a,b) was deduced from the intensity profile on the DNA loop *before* the encounter between roadblock and the loop. The intensity of a single loop (i.e. I_2x) and the predicted putative double loop (I_4x) is shown in the lines in Fig. 5c. We observe that the maximum intensity of the DNA equals the value of the intensity I_2x of a *single* loop while it never comes close to the predicted doubled loop intensity I_4x at any stage during loop extrusion at and after the encounter. From these side-flow visualizations, we thus did not obtain any indication of a doubled loop intensity (e.g. Figure 5a–g)—in contrast to the prediction of the pseudotopological model. Our data are therefore not supporting the key predictions made by the pseudotopological model, but instead support the nontopological model.

#### Test of prediction 2

The hold-and-feed model predicts that roadblocks will reside near the stem of the loop. This is, however, not observed. Instead, soon after the encounter, the roadblock is observed to be located within the extruded loop and separated from condensin or cohesin, see main figures from Pradhan et. al.^10^ and Fig. 5a,f,h,i.

**Figure 6:**
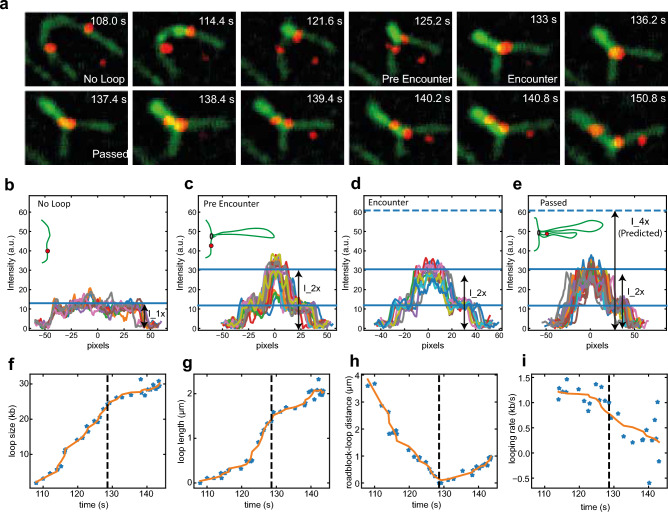
Test of prediction 2 associated with the pseudotopological model. (**a**) Snapshots of DNA (green) and a 39 nm-wide roadblock (red) with sideflow showing the continuous approach of the roadblock toward the loop stem before encounter (time points until 128 s) and the continuously increasing distance between roadblock and loop stem after encounter. (**b–e**) Loop intensities during the time series. (**f**) Loop size (kb) versus time. (**g**) Loop length (μm) versus time. (**h**) Distance (μm) between roadblock and loop stem versus time. (**i**) Looping rate versus time. Dashed lines in (**f–i**) shows the time of encounter. λ-DNA and a gold nanoparticle of 39 nm was used in all panels.

We can illustrate this in more detail by an example given in Fig. 6. Here, the loop contained 24 kb of DNA before encounter of the roadblock. Upon encounter, the loop-extrusion speed did not abruptly change but loop extrusion continued monotonously at an approximately constant rate until the loop disrupted when it reached its final size of 30 kb. For this example, Shaltiel et al*.* predict the appearance of a second loop (loop-2) of 6 kb. However, no increased intensity is observed close to the stem of the loop, see Fig. 6b–e. The pseudotopological model of further predicts that the roadblock initially resides at the stem of the loop until the cumulative drag force on loop-2 and roadblock exceeds the drag force on loop-1. Note that the drag force on the 24 kb-long loop-1 is very large, ~ 0.65 pN (Fig. 3d), while the drag forces on the fully extruded loop-2 remain low at ~ 0.16 pN and the drag force on the particle is estimated at ~ 0.03 pN (Fig. 3b–d). Hence, one does not expect any slippage of loop-1 into loop-2. The roadblock should therefore, according to the pseudotopological model, reside at the loop base while the loop continues to grow. However, the roadblock was experimentally observed to clearly travel continuously (and not suddenly as predicted for a potential slipping event) along one arm of the extruded loop (Fig. 6a–h). Quantitatively, after the encounter, the extruded loop enlarged from 1.6 µm to 2.1 µm under sideflow conditions (due to its increase from 24 to 30 kb). Each arm of the extruded loop in the final state thus contains 15 kb (half of the entire loop). For continuous loop extrusion into one loop, one would thus expect the particle to have moved to a distance of 6 kb/15 kb $\cdot$ 2.1 µm = 0.84 µm from the loop stem, which indeed is in excellent agreement with measurements of the ~ 0.9 µm distance between roadblock and loop stem (Fig. 6h). Yet another measure is to consider whether or not the particle moved into the loop with a speed comparable to the loop-extrusion speed. This would be expected in our scenario of continuous loop extrusion where the particle smoothly moves into the extruded loop, whereas the pseudotopological model would predict the particle to stall at the loop stem and then suddenly speed up in a slippage event. Experimentally, we observed that the particle moved over a distance of ~ 0.9 µm in 13 s, i.e. with a speed of ~ 0.07 µm/s. This fits well with the loop-extrusion speed which for this example is ~ 0.08 µm/s (since it is ~ 0.5 kb/s (Fig. 6i) and the 6 kb is extruded into 0.9 µm along one arm of the loop). We thus observe that the loop-extrusion speed is the same as the velocity with which the roadblock moves away from loop base.

The data thus are in full accordance with continuous extrusion of the DNA and the particle into one loop, as predicted by the nontopological model, while the data are not in accordance with the pseudotopological model.

#### Test of prediction 3

How do the size of the extruded DNA loop and the MSD of the particle correlate? We previously reported (Figs. 1, 2, 3 and 4 of Ref.^10^) that we observed a strong correlation between an increase in loop size and an increase in MSD after the encounter with the roadblock. Here, we focus more in detail on stalling and passing events, where the MSD remained constant, and we searched for data that were compatible with prediction 3. In the model prediction, the loop size can grow while the MSD of the particle may stay constant (Fig. 4a). Note also that here we discuss experiments without an applied side flow. Figure 4b shows an example of what we called a stalling event where the MSD (black) remained constant for more than 10 s. Clearly, the corresponding loop sizes during the period when the MSD was constant (e.g. between 30 and 60 s in Fig. 4b and above 30 s in Fig. 4c) was also found to be constant. This is the typical behavior. We examined all our stalling events (N = 15), and we found that *none* of the events showed a concomitant increase in loop size (see Fig. 4d for additional examples). The observation that the loop size *never* increased while the MSD remained constant is not consistent with one of the key predictions made by the pseudotopological while it meets the expectation of the nontopological model.

## Conclusion

We conclude that the predictions of the pseudotopological models by Shaltiel et al*.* and Nomidis et al*.* are not consistent with our experimental observations. Instead, our experimental data are fully consistent with a nontopological model of loop extrusion.

While the loop extrusion mechanism remains unresolved and currently a matter of strong debate, a nontopological model for condensin and cohesin can be envisioned, as outlined by Oldenkamp and Rowland^8^. The high structural similarity between condensin and cohesin (similar length of the SMC arms and the kleisin, presence of both HEAT-A and HEAT-B) suggests that the underlying loop extrusion mechanism is highly analogous. It is unclear if SMC5/6, which has been observed to dimerize for loop extrusion in vitro^15^ and whose kleisin is almost half shorter than for condensin (402 amino acids for yeast Nse4 versus 754 amino acids for yeast Brn1), also uses a similar mechanism. The available crosslink data by Shaltiel et al*.* for yeast condensin allow both a pseudotopological and a nontopological DNA entrapment. In the nontopological version, the HEAT-A-bound N-terminal fragment of the kleisin could flip open, alleviating particle bypass through the HEAT-A compartment. Upon ATP hydrolysis, the ATP heads disengage^16^, allowing roadblocks to pass through the ATP heads, see Fig. 1b (more details of this model in Dekker, Haering, Peters, Rowland, submitted).

Models such as these can be further tested in future experiments that probe the traversal of large roadblock proteins that symmetrically embrace DNA. Small (~ 10 nm diameter) roadblocks such as nucleosomes can be passed by SMC complexes directly, as distances of up to tens of nm between the ATPase heads have been observed in AFM images of condensin and cohesin^16,17^. Roadblocks of any size which bind to DNA asymmetrically on one side (like the dCas9-nanoparticle constructs used in this study) can pass as long as their DNA-binding linker is long enough. However, this scenario could differ for a symmetric protein complex that fully wraps around DNA, like for example the MCM hexamer (a part of the eukaryotic replication machinery). Roadblock passage experiments with such DNA-enclosing roadblocks would be of interest to test this prediction.

## Materials and methods

### Data collection

Data acquisition and analysis of loop extrusion and roadblock passages was performed as described in detail in Ref.^10^.

### Estimation of the flow velocity

Occasionally, bright autofluorescent particles and/or free fluorescently labeled nanoparticles (e.g. after breakage of dsDNA or rare dissociation of dCas9 from DNA) were observed flowing through a particular field of view under buffer flow. These particles were detected and tracked for as long as they were present in the field of view (Fig. S1). The distance between successive localizations within one field of view were converted to the instantaneous flow speed using the imaging interval. Since the images were recorded using Highly Inclined and Laminated Optical sheet (HILO) microscopy^10^, the recorded fluorescence of fly-by particles which were in focus are localized within a distance of about approximately a micrometer from the axial plane in which DNA is imaged such that the flow speed encountered by fly-by particles and roadblocks on DNA or the DNA itself were comparable.

### Estimation of the drag force

The drag force on a spherical particle is computed according to Stokes’ law ${F}_{drag}=6\pi \eta Rv$, where $\eta =1~mPa s$ for a water-based buffer, $R$ is the particle radius, and $v$ the buffer flow speed. The drag force on the extruded DNA loop is estimated based on Ref.^13^. Pedersen et al*.* compute the parallel and perpendicular drag coefficient per unit length on a cylinder of radius 1 nm and obtain values in the range 2 – 7 $\cdot$ 10^−3^ pN s µm^−2^ numerically and from fits to experimental data. Since the majority of the DNA loop is aligned with the buffer flow in our experiments, we consider the lower bound of these estimates, i.e. $\gamma =1\cdot {10}^{-3}$ pN s µm^−2^ per unit length. The drag force on the extruded DNA loop is computed as ${F}_{drag}^{loop}=\gamma v{L}_{c}$ where ${L}_{c}$ is the contour length of a strand of DNA of number of basepairs N, i.e. ${L}_{c}=\alpha N\cdot 0.34~nm$, where 0.34 nm is the distance between basepairs, and $\alpha$ is a factor which accounts for a slight DNA stretching when using intercalating dyes^18^ like Sytox Orange as in Ref.^10^.

### Measurement of DNA intensity profiles in side flow images

Image frames were marked as “No Loop” when loop was not yet initiated; “Pre Encounter” when a loop was growing but the roadblock had not yet encountered (colocalized) with the stem of the loop; “Encounter” when the roadblock and stem of the loop colocalized; and “Passed” when “roadblock” was observed inside the loop. Contour lines were manually drawn on the DNA and intensity profiles were obtained using imageJ^19^. Line profiles from each group were plotted together in Python.

### Quantification of loop length, size, distance to roadblock, and loop extrusion rate in side flow experiments

Intensities inside and outside the loop were extracted by manually drawing rectangular boxes with width of 11 pixels. DNA loop size was calculated as 48.5 kb · (I_loop_/I_total_). The roadblock-loop distance was calculated by manually drawing a line between the roadblock and the stem of the loop and converting the length of the line from pixels to µm with pixel size of 0.108 µm/pixel. Similarly, loop length was calculated by drawing a line from the stem of the loop to the end of the loop and image frames with loops in the optical focus were chosen for length calculations.

## Supplementary Information

Supplementary Figure S1.

## Data Availability

Custom code and data used for the analysis are deposited under https://doi.org/10.5281/zenodo.6959501.
