# Author Correction: Testing pseudotopological and nontopological models for SMC-driven DNA loop extrusion against roadblock-traversal experiments

**DOI:** 10.1038/s41598-023-38687-5

**Published:** 2023-07-18

**Authors:** Roman Barth, Biswajit Pradhan, Eugene Kim, Iain F. Davidson, Jaco van der Torre, Jan‑Michael Peters, Cees Dekker

**Affiliations:** 1 Department of Bionanoscience, Kavli Institute of Nanoscience Delft, Delft University of Technology, Delft, The Netherlands; 2 Research Institute of Molecular Pathology (IMP), Vienna Biocenter (VBC), Vienna, Austria; 3 Present Address: Max-Planck Institute of Biophysics, Frankfurt Am Main, Germany

Correction to: *Scientific Reports* 10.1038/s41598-023-35359-2, published online 19 May 2023

The original version of this Article contained an error in Figure 1b-1, where the fore- and background order of the strands “DNA” (in black) and “Brn1 Kleisin” (in green), were switched.

The original Figure 1 and accompanying legend appear below.

**Figure 1:**
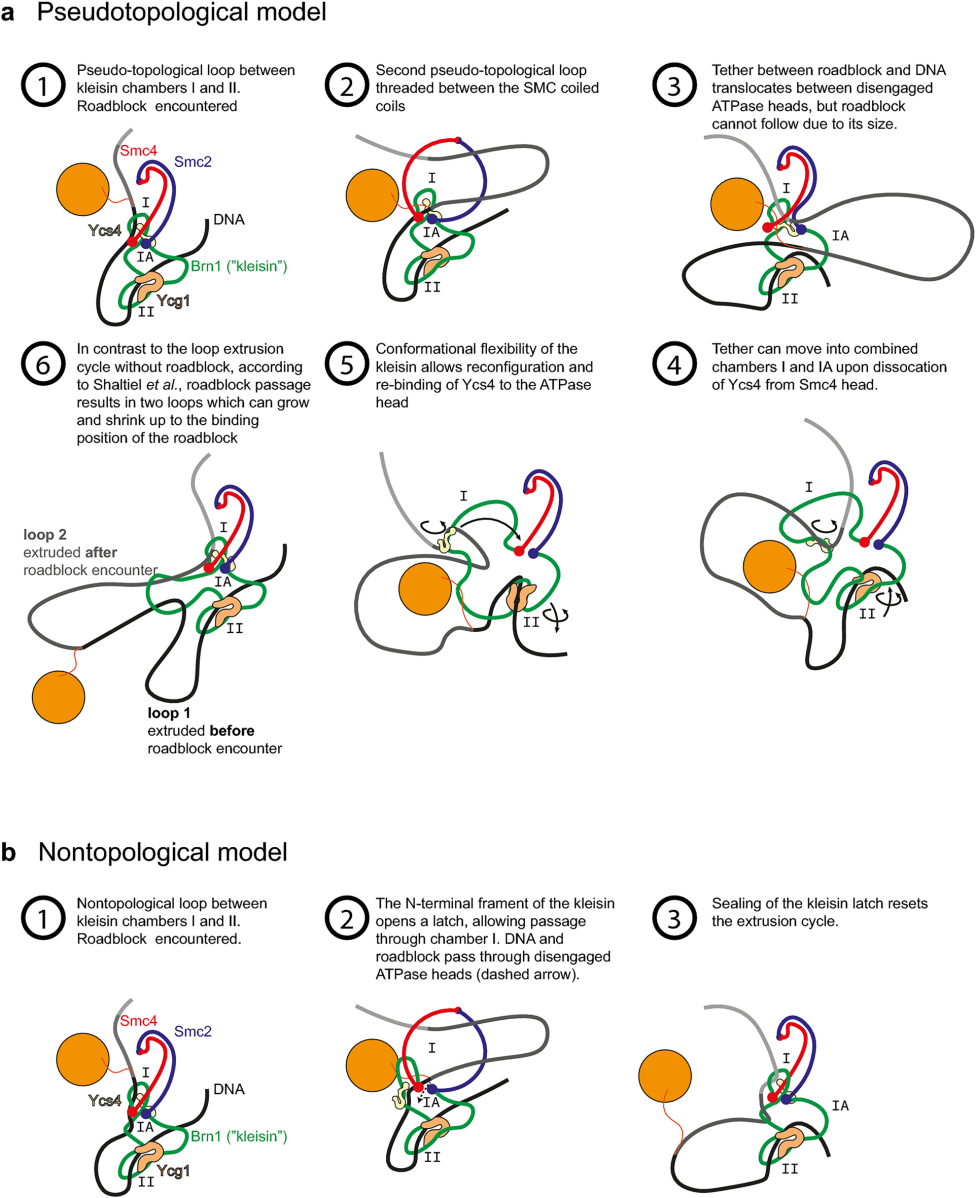
Description of the mechanism postulated by Shaltiel et al. for roadblock passage into an extruded loop on the DNA and a potential nontopological model. (**a**) The steps through the proposed DNA loop extrusion cycle are commented in more detail in steps 1–6 within the figure. Adapted from Ref.^11^. (**b**) Potential nontopological model which is closely analogous to the pseudotopological model, but with a slight variation in the DNA-SMC topology which allows particle bypass.

The original Article has been corrected.

