# Supplementary material for: Testing pseudotopological and nontopological models for SMC-driven DNA loop extrusion against roadblock-traversal experiments
Source: Sci Rep. 2023 May 19;13:8100. doi: 10.1038/s41598-023-35359-2 (PMC10199080; doi:10.1038/s41598-023-35359-2)
Supplement: Supplementary file 1 — Supplementary Figure S1. [file 41598_2023_35359_MOESM1_ESM.pdf]

Supporting Information

Testing pseudotopological and nontopological models for SMC-driven DNA loop extrusion against roadblock-traversal experiments

Roman Barth[1,$], Biswajit Pradhan[1,3,$], Eugene Kim[1,3], Iain F. Davidson[2], Jaco van der Torre[1], Jan-Michael Peters[2], and Cees Dekker[1,*]

[1] Department of Bionanoscience, Kavli Institute of Nanoscience Delft, Delft University of Technology, Delft, Netherlands

[2] Research Institute of Molecular Pathology (IMP), Vienna Biocenter (VBC), Vienna, Austria.

[3] Current address: Max-Planck Institute of Biophysics, Frankfurt am Main, Germany

[$] Equal contribution

* Corresponding author; email c.dekker@tudelft.nl

This PDF file includes:

Figure S1

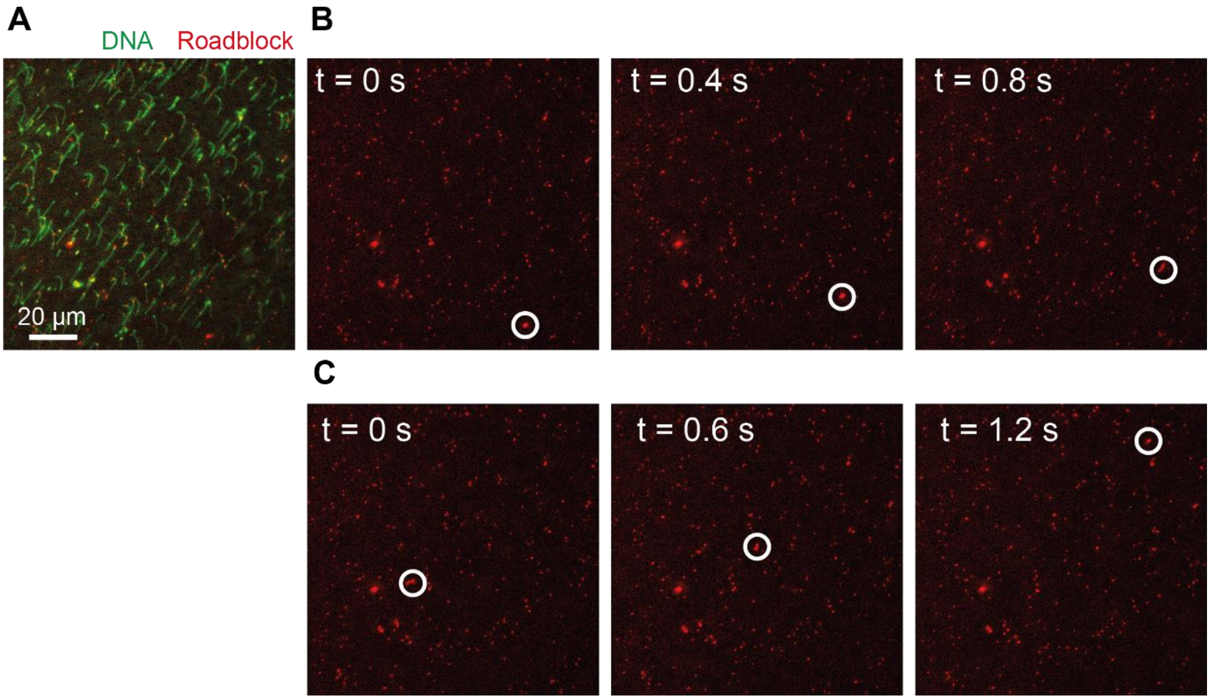

Figure S1: Measurement of the flow speed using unattached fluorescent particles. (A) A field of view is shown in which a fluorescently labelled roadblock (red) is attached to a Sytox Orange-stained DNA (green). The DNA is stretched by a buffer flow perpendicular to the DNA's end-to-end vector. **(B-C)** Two instances of free fluorescent particles (marked by a white circle) are shown that cross the field of view within 0.8 and 1.2 s, respectively. The flow speed is computed from the distance between localizations in successive frames.
